# Development and Structural Characterization of Pullulan/Lecithin/Zein Composite Nanofibers Loaded with Mountain Germander (*Teucrium montanum*) Polyphenolic Extract

**DOI:** 10.3390/foods14213619

**Published:** 2025-10-23

**Authors:** Ana Mandura Jarić, Darija Domazet Jurašin, Predrag Petrović, Sunčica Kuzmić, Laura Nižić Nodilo, Aleksandra Vojvodić Cebin, Danijela Šeremet, Draženka Komes

**Affiliations:** 1 Department of Food Engineering, Faculty of Food Technology and Biotechnology, University of Zagreb, Pierotti St 6, 10 000 Zagreb, Croatia; ana.mandura.jaric@pbf.unizg.hr (A.M.J.); aleksandra.vojvodic@pbf.unizg.hr (A.V.C.); danijela.seremet@pbf.unizg.hr (D.Š.); 2 Department of Physical Chemistry, Ruđer Bošković Institute, Bijenička 54, 10 000 Zagreb, Croatia; djurasin@irb.hr; 3 Department of Chemical Engineering, Faculty of Technology and Metallurgy, University of Belgrade, Karnegijeva St 4, 11 000 Belgrade, Serbia; 4 Forensic Science Centre “Ivan Vučetić” Zagreb, Forensic Science Office, Ilica St 335, 10 000 Zagreb, Croatia; skuzmic@mup.hr; 5 Department of Pharmaceutical Technology, Faculty of Pharmacy and Biochemistry, University of Zagreb, Domagojeva St 2, 10 000 Zagreb, Croatia; lnizic@pharma.hr

**Keywords:** encapsulation, electrospinning, nanofibers, pullulan, zein, *Teucrium montanum*

## Abstract

In this study, the electrospinning technique was employed to encapsulate mountain germander (MG) polyphenolic extract into pullulan/zein (PUL:ZE) delivery systems stabilized with sunflower lecithin. The rheological and physical properties of the pullulan (PUL), PUL:ZE, and zein (ZE) polymer solutions were evaluated to assess their electrospinnability potential. Fabricated nanofibers were then characterized for their morphology, physicochemical, and thermal properties, as well as encapsulation efficiency and simulated in vitro digestion. The elastic component of the polymer solution, quantified by the Deborah number, showed a strong correlation with nanofiber diameter (r = 0.75). FT-IR spectra confirmed the role of sunflower lecithin as a mediator in the formation of hydrogen and hydrophobic interactions among PUL, ZE, and polyphenols. The circular dichroism spectra confirmed the influence of the MG extract on the change in the secondary conformation of the protein structure. The PUL:ZE delivery matrix proved to be suitable for the retention of phenylethanoid glycosides (encapsulation efficiency > 73%). The formulation 50PUL:50ZE was found to have the highest potential for prolonged release of polyphenols under gastrointestinal in vitro conditions. These findings propose a water-based electrospinning approach for designing polyphenolic delivery systems stabilized with lecithin for potential applications in active food packaging or nutraceutical products.

## 1. Introduction

Valorization of herbal extracts as a source of bioactive compounds in food products and nutraceuticals is progressing rapidly due to the increasing global demand for functional foods and dietary supplements [1]. Because bioactive compounds (BCs) are susceptible to degradation under certain conditions (e.g., temperature, pH, storage, oxidation processes), investment is required in technological solutions to protect BCs, retain their health-promoting effects, and improve their bioavailability.

These challenges can be effectively addressed through nanoencapsulation, an advanced technology designed to protect and stabilize bioactive compounds by entrapping them within a carrier matrix or shell, resulting in nanoscale delivery systems such as particles, films, micelles, dispersions, or fibers [2]. Recently, electrospinning has gained considerable attention as a promising technique for producing ultrafine fibers from polymer liquid solutions in sheet-like structures with adjustable diameters in the nano- to micrometer range. This technique involves an electrohydrodynamic fiber formation process in which a polymer droplet is stretched into a beam under a high-voltage electric field. The proper formation of a Taylor cone, a prerequisite for a stable polymer beam and suitable production of electrospun nanofibers, is greatly influenced by simultaneously optimized process, environmental, and solution parameters [3]. The distinctive properties of fiber-formed sheets include a high specific surface area, high porosity, tunable wetting behavior, and high specificity in the nanofibers’ alignment within the sheet itself (crosslinked, parallel, random, hoop-like pattern, etc.), enabling efficient integration, stabilization, and controlled release of BCs [4,5]. Therefore, the possibility of targeted morphological, mechanical, and structural functionalization by selecting the solvent type, polymer matrix (molecular weight, concentration), or processing configuration (setup with single, coaxial, or multi-fluid solutions) makes electrospinning a cutting-edge technology for state-of-the-art applications in biosensors [6], advanced drug delivery systems [7], and biomedicine [8]. Recently, the application of electrospun fibers in the food industry has attracted significant interest in active food packaging with incorporated antimicrobial and antioxidant agents [9], fabrication of ethylene absorbent material to extend shelf life [10], enzyme immobilization for improved stability and efficiency [11], and encapsulation of omega-3 fatty acids and probiotics [12,13], while there are no studies investigating the potential of direct incorporation of electrospun fibers into the food matrix so far.

The biodegradability and non-toxicity of the polymers used are general requirements that must be fulfilled for the application of electrospun delivery systems in food-related areas. Most are derived from biological sources (e.g., polylactides, polyols, synthesized polysaccharides, cellulose acetate) or occur naturally (e.g., alginate, starch, cellulose, gum arabic) [14]. Pullulan (PUL) is often chosen as an electrospinning biopolymer due to its odorless and tasteless nature, high thermal stability, and relative resistance to digestive enzymes, making it suitable for encapsulation [15,16]. It is a non-gelling, linear, hydrophilic polysaccharide synthesized from *Aureobasidium pullulans*. In pharmaceutical applications, PUL is often combined with other bio-based polymers to optimize viscosity and enhance formulation stability, e.g., with carboxymethyl starch for thymol delivery [17], with pea protein for allicin delivery [18], and with zein for carvone encapsulation [19]. Proteins also offer a promising alternative due to their amphiphilic character and ability to bind polyphenols through multiple interaction sites, potentially modifying secondary structures to benefit the structural properties of delivery systems [20]. Zein, a protein fraction derived from cornmeal, has gained popularity in research as a multifunctional biopolymer owing to its GRAS (“generally recognized as safe”) status, high hydrophobic amino acid profile (>50%), film-forming capacity, amphiphilic characteristics, and intrinsic self-assembly behavior [21] Previous research on the valorization of zein indicates strong potential for its application as a substrate for the formulation of nanocomposite films for wound healing [22], active coating [9], and delivery systems for, e.g., savory essential oil [23], pomegranate peel extract [24], and curcumin [25]. However, in electrospinning, the use of zein alone often results in poor spinnability, inconsistency in fiber morphology, low mechanical strength, and needle clogging [26,27]. Compared with crosslinking methods, which generally employ toxic crosslinking agents, combining natural and/or synthetic polymers with zein through hybrid electrospinning is a non-toxic and suitable method to overcome the aforementioned drawbacks regarding zein’s application in food-related matrices. Positive effects of incorporating hydrophilic polysaccharides, such as pectin [28], carrageenan [29], sodium alginate [30], and gum arabic [31], into zein solutions have been reported in terms of improved stability and delivery capabilities of the formulated nanoparticles. In addition, recent studies investigating the interaction between lecithin and zein in the form of nanoparticles have attracted attention due to the promising potential of this natural surfactant to enhance the physicochemical stability and gastrointestinal bioavailability of *Monascus* yellow pigment [32], flavonoids from *Smilax glabra* [33], anthocyanins [34], and others. Lecithin, as an amphiphilic natural surfactant composed of various phospholipid types, has proven to be a promising stabilizing agent for zein composite emulsions with hydrophilic pectin [28] and zein-based solutions [9,35] by forming hydrogen bonds, electrostatic interactions, and hydrophobic effects, as well as inducing changes in the secondary structure. However, interactions among zein, lecithin, and pullulan have not yet been investigated, and the structural and functional characteristics of zein–pullulan composite nanofibers previously stabilized with lecithin remain unknown.

*Teucrium montanum* L., known as mountain germander, is a Mediterranean medicinal plant traditionally used to treat digestive and respiratory diseases, strengthen immunity, and promote blood purification [36,37,38,39]. Its phenolic composition is dominated by flavonoids, hydroxybenzoic acids, and hydroxycinnamic acids, while phenylethanoid glycosides (PGs) remain relatively unexplored. Phenylethanoid glycosides (PGs) represent an important group of bioactive compounds prevalent in numerous medicinal plant species used in traditional Chinese medicine. Structurally, they are characterized by a β-glucopyranose unit bonded to a phenethyl alcohol via a glycosidic linkage [40]. This central phenylethanol glucoside skeleton is often substituted with phenolic acids and sugars through ester or additional glycosidic bonds. Recent findings highlight PGs’ remarkable range of biological activities, such as anti-estrogenic function [41], ultraviolet protection [42], and anti-inflammatory [43], antiviral [44], and neuroprotective [45] effects, etc. To date, available studies have confirmed the degradation susceptibility of PGs under changes in pH, light exposure, and higher temperature regimes (50–80 °C), probably due to the presence of phenolic hydroxyl groups and the tendency for cis-/trans-conformation change [46].

The aim of this study was to explore the electrospinnability potential of a PUL/ZE carrier matrix stabilized with lecithin for the encapsulation of MG extract via blending electrospinning. We also investigated how the zein ratio influences the viscoelastic properties of polymer solutions, morphology, physicochemical properties (modifications in the ATR-FT-IR spectra and zeta potential), structural changes in protein conformation, and thermal stability of nanofibers, as well as their encapsulation efficiency and in vitro release performance. This study introduces a green strategy for developing PG-rich delivery systems based on a biopolymer composite matrix, offering prospects for use in active packaging and nutraceutical applications.

## 2. Materials and Methods

### 2.1. Material

Mountain germander (*T. montanum* L.) was purchased from the local supplier (Ljekovito bilje j.d.o.o, Šibenik, Croatia). Plant material was collected at the municipality of Kistanje; Šibenik-Knin County (Adriatic region of Croatia, August 2020). Milled and sieved areal parts of the plant (<450 μm) were used in all experiments. Voucher specimen (ID: 75518) was deposited in the Herbarium Croaticum (Faculty of Science, Croatia).

### 2.2. Chemicals and Reagents

Pullulan (food grade, average Mr: 150 kDa, CAS: 9057-02-7) was supplied from Biosynth s.r.o. (Bratislava, Slovakia). Sunflower lecithin (Nutrimedica, EU) was supplied from the local store Terra Organica (Zagreb, Croatia). Purified zein was purchased from Acros Organics (Thermo Fisher Scientific, Waltham, MA, USA).

All chemicals and reagents used in the experiments were of analytical and HPLC grade. Folin–Ciocalteu reagent, potassium chloride, sodium hydrogen carbonate, potassium hydrogen phosphate, magnesium chloride hexahydrate, sodium chloride, ammonium carbonate, sodium hydroxide, and glacial acetic acid were purchased from Kemika d.d. (Zagreb, Croatia). Anhydrous sodium carbonate was supplied by Gram-Mol d.o.o. (Zagreb, Croatia). Methanol, acetonitrile, hydrochloric acid (37% *v*/*v*), orthophosphoric acid (85% *v/v*) sodium hydroxide, and formic acid were purchased from Carlo Erba Reagents (Val de Reuil, France). Secondary analytical standards, i.e., echinacoside (CAS: 82854-37-3, >98% purity) and verbascoside (CAS: 61276-17-3, >98% purity), were provided by Biosynth s.r.o. (Bratislava, Slovakia). Pepsin from porcine gastric mucosa (474 U mg^−1^ protein, CAS: 9001-75-6), pancreatin from porcine pancreas (4*USP, CAS: 8049-47-6), and bile bovine salts (unfractionated, CAS: 8008-63-7) were purchased from Sigma Aldrich (St. Louis, MI, USA).

### 2.3. Characterization of Mountain Germander Polyphenolic Extract

#### 2.3.1. Extract Preparation

Previously optimized heat-assisted extraction parameters (1 g:100 mL, mass to solvent ratio, 100 °C, 30 min) were employed for the preparation of polyphenolic aqueous extract [47]. Filtered extract (Whatman Grade 1) was concentrated 20-fold on a rotary evaporator (RV 8, IKA, Staufen, Germany) and further diluted with glacial acetic acid (1:1 *v*/*v*).

#### 2.3.2. Determination of Total Phenolic Content (TPC)

Total phenolic content was determined by Singleton and Rossi (1965) [48]. The results were calculated using a standard calibration curve for echinacoside (50–1000 µg mL^−1^), and were expressed in mg of echinacoside equivalents per liter of extract (mg eq. ECH L^−1^) (Table 1).

#### 2.3.3. HPLC-UV-DAD Quantification of Phenylethanoid Glycosides (PGs)

Identification of polyphenolic compounds was carried out according to our previous study [49]. Chromatographic separation was performed on a Zorbax extended C-18 analytical column (L × I.D. 250 × 4.5 mm, 5 µm particle size) (Agilent Technologies, Santa Clara, CA, USA) using gradient elution (Mobile Phase A: 1% formic acid in water, *v*/*v*; Mobile Phase B: 1% formic acid in acetonitrile, *v*/*v*), over 52 min with an additional 10 min equilibration. The content of Mobile Phase B was 0–5 min, 3%; 5–45 min, 40%; 45–47 min, 70%; 47–52 min, 70% B. The method parameters were set as follows: flow rate, 1 mL min^−1^; temperature, 25 °C; volume of the injected sample, 5 µL. The UV spectra of eluted compounds were recorded in the range of 260–370 nm by DAD (Agilent Technologies, Santa Clara, CA, USA). Quantification of PGs, as the most represented polyphenols in the analyzed mountain germander extract, was carried out by standard calibration curves for echinacoside and verbascoside, with maximum UV absorption at 320 nm. Results for teupolioside, stachysoside A, and poliumoside were expressed in mg of echinacoside equivalents per liter of extract (mg eq. ECH L^−1^). Identified PGs, i.e., echinacoside, teupolioside, stachysoside A, poliumoside, and verbascoside, accounted for approximately 66% of the total phenolic content (Table 1).

### 2.4. Preparation of Solutions and Electrospinning Performance

To overcome the challenges of encapsulation by water-based electrospinning in terms of the limited selection of biopolymers, the requirement for green solvents, and fast dissolution of hydrophilic polymers in aqueous medium, polymer solutions (15% of total polymer content, *w*/*w*) were prepared in acidified polyphenolic extract concentrated 10-fold with respect to the defined ratio of pullulan or/and zein (Table 2). The content of sunflower lecithin as a stabilizing agent was fixed for all formulations (3% *w*/*w*). Complete homogenization of the polymer solutions (carrier matrix + stabilizer + polyphenolic extract) was carried out at room temperature for 24 h on a magnetic stirrer (SMHS-6, Witeg Labortehnik GmbH, Wertheim, Germany).

**Figure 1:**
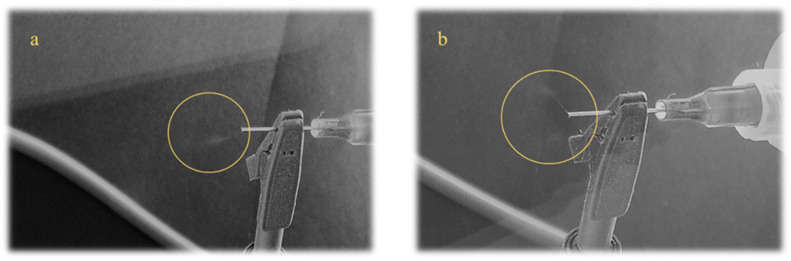
Visualization of the electrospinning process showing: (**a**) formation of the Taylor cone and a stable, elongated polymer jet, and (**b**) formation of the Taylor cone followed by jet destabilization in multiple directions due to unsuitable processing conditions or rheological properties of the polymer solution. Taylor cone is marked within the yellow circle.

**Figure 2:**
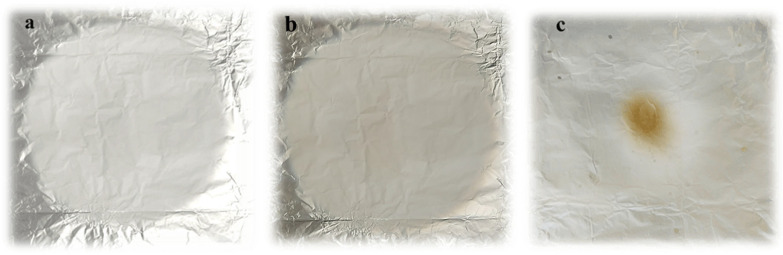
Examples of successfully fabricated nanofiber sheets: (**a**) 50PUL:50ZE and (**b**) 20PUL:80ZE. (**c**) Visual appearance of failed electrospinning from the 100ZE solution as the result of electrospraying dominance.

Blending electrospinning of polymer solutions was performed using an electrospinning device from Spinbox Systems (Bionicia, Valencia, Spain) under the following conditions: flow rate, 0.8 mL h^−1^; high positive voltage, 20–23 kV; distance between the collector and the needle tip, 14–15 cm. The needle size was 22 gauge. The process was carried out at temperatures and relative humidity between 25–28 °C and 30–35%, respectively. Fiber samples were collected on a plate collector covered with aluminum foil, lyophilized for 24 h to remove acetic acid residues (Christ, ALPHA 1–2 Ldplus, Osterode, Germany), and stored in the exicator at room temperature until the analysis. A visualization of the electrospinning process and examples of successfully and unsuccessfully produced nanofiber mats are presented in Figure 1 and Figure 2.

### 2.5. Physical Properties of Polymer Solutions

The *dry matter content* of polymer solutions for the calculation of encapsulation efficiency was analyzed using the AOAC standard method [50].

The *surface tension* of previously tempered solutions (25 °C) was analyzed on a tensiometer KRÜSS K100 (A.KRÜSS Optronic GmbH, Hamburg, Germany) using the standardized Du Noüy ring method. Calibration measurements were conducted with Milli-Q water at 25 °C.

*The electrical conductivity* of all tested samples was measured with a conductometer (Lab 945 analog conductivity benchtop meter, Xylem Inc., Washington, DC, USA) at 25 °C.

*Density measurements* of the polymer solutions were performed using a digital density meter (DMA 1001, Anton Paar GMBH, Graz, Austria). Calibration measurements, as well as analyses of all samples, were performed with distilled water at 25 °C.

Experimentally obtained values of density and surface tension were further used for the calculation of nondimensional numbers, namely the *Ohnesorge number* (*Oh*) for tested polymer solutions which resulted in nanofiber structure, while, additionally, the *Deborah number* (*De*) was calculated for all polymer solutions exerting relaxation times determined by frequency sweep tests. *Oh* was calculated using Equation (1), where µ is the apparent viscosity (Pa), *ρ* is the density of polymer blends (kg m^−3^), *σ* is the surface tension (N m^−1^), and *L* is the gauge diameter (m).

(1) $$Oh=\frac{µ}{\sqrt{\rho \sigma L}}$$

Since electrospinning represents a capillary inertial process, capillary-inertial time, which incorporates *ρ*, *µ*, and *L*, is included in the calculation of the *De* number instead of an arbitrary process time scale. Thus, *De* was calculated using Equation (2), where *τ* is the relaxation time determined from frequency sweep test for each polymer solution, *ρ* is the density of polymer blends (kg m^−3^), *σ* is the surface tension (N m^−1^), *L* is the gauge diameter (m), and *µ* is the apparent viscosity (Pa).

(2) $$De=\frac{\tau }{\sqrt{\frac{\rho L^{3}}{\mu }}}$$

### 2.6. Viscosity Curve and Rheological Tests of Polymer Solutions

Rheological measurements of the formulated polymer solutions were performed on a Modular Compact Rheometer (MCR102, Anton Paar GmbH, Graz, Austria) equipped with an integrated air-cooled Peltier temperature control system. All data were calculated by the rheometer’s software (RheoCompass TM Light, Anton Paar GmbH, Graz, Austria).

*Viscosity curve determination* was performed by a rotational test using a cone (1°)–plate measuring system (CP50), with the gap set at 0.102 mm. All samples were equilibrated at 25 °C for 3 min. The shear dependence of viscosity was measured in the range of 0.1–100 s^−1^.

*Oscillatory tests* were conducted by a parallel-plate (PP50) measuring system. The *amplitude sweep test* was performed in a strain-controlled mode at a 6.28 rad s^−1^ angular frequency. The range of shear strain applied was 0.1–100% with the gap set to 0.500 mm. The *frequency sweep test* was performed in an angular frequency range of 0.1–100 rad s^−1^ at 0.5% strain deformation selected from the previously defined LVE range (gap: 0.500 mm). For both tests, the samples were previously equilibrated at 25 °C for 3 min.

### 2.7. Characterization of Electrospun Nanofibers

#### 2.7.1. Scanning Electron Microscopy (SEM) Analysis

Morphological characterization, as well as the diameter of the nanofibers, were analyzed by scanning electronic microscopy (SEM) on a TESCAN Mira3 microscope (Tescan Group, Brno, Czech Republic). Samples were sputtered with a gold layer prior to microscopic analysis to ensure electrical conductivity. Scanning was evaluated under 15 kV.

The *relative distribution* of the nanofibers’ diameter was evaluated by combining ImageJ 1.53 software for the analysis of SEM micrographs to determine nanofibers’ diameter, and Origin Pro 8.0 software for graphing and data analysis of the measured diameters (OriginLab Corporation, Northampton, MA, USA). Gaussian fit was applied for the peak of each histogram.

#### 2.7.2. Attenuated Total Reflectance Fourier Transform Infrared Spectroscopy (ATR-FT-IR) Analysis

ATR-FT-IR analysis (Nicolet iS10, Thermo Scientific, Waltham, MA, USA) was employed for evaluating the interactions between functional groups of polymers and extract in the electrospun nanofibers. A total of 32 cumulative scans were taken in the frequency range of 400–4000 cm^−1^, with a resolution of 0.16 cm^−1^ to obtain absorption spectra.

#### 2.7.3. Circular Dichroism (CD)

CD spectra were acquired on a Jasco J-815 spectropolarimeter (Jasco Corporation, Tokyo, Japan) using a liquid sandwich quartz cell (l = 0.1 mm). Samples (1 mg mL^−1^) were dissolved in 10 M orthophosphoric acid. The parameters were set as follows: spectral range between 190–350 nm; spectra accumulation, 3; resolution, 0.2 nm; bandwidth, 2 nm; scanning speed, 200 nm min^−1^; response, 1 s.

#### 2.7.4. Differential Scanning Calorimetry (DSC)

Differential scanning calorimetry (DSC) was employed for evaluation of the thermal properties of the tested nanofibers delivery systems under a selected temperature range using a Mettler Toledo DSC823e measuring module (Mettler Toledo, Greifensee, Switzerland). Each sample (2–3 mg) was hermetically sealed in an aluminum pan and analyzed in one heating cycle under an inert atmosphere (nitrogen flow: 50 mL min^−1^).

Each cycle comprised three segments: (i) cooling from 25.0 to −70.0 °C at −30.00 °C min^−1^, (ii) isothermal −70.0 °C for 3.0 min, and (iii) heating from −70.0 to 250 °C at 10 °C min^−1^.

#### 2.7.5. Encapsulation Efficiency (%)

The encapsulation efficiency (EE) of TPC [48] and individual PGs was determined by Equation (3)

(3) $$IU\left(\%\right)=\frac{W_{nf}}{W_{fs}}\times 100$$

where *W_nf_* represents the content of determined TPC or individual PGs on the dry matter of nanofibers, and *W_fs_* is the content of determined TPC or individual PGs in the initial liquid polymer solution. The dry matter content of lyophilized nanofibers for the calculations was set at 100%.

#### 2.7.6. Zeta Potential

To predict the colloidal stability of the formulated nanofibers in the acidified suspension, zeta potential was measured. Firstly, each weighted sample (1 mg) was mixed with 1 mL of 10% glacial acetic acid (*v*/*v*). The undiluted samples were then placed in an ultrasonic bath for 15 min at 30 °C and the resulting suspensions were analyzed at 25 °C on a Malvern Zetasizer Ultra (Malvern Panalytical, Malvern, UK) in triplicate using a DTS1070 cuvette.

#### 2.7.7. In Vitro Digestion

In vitro static digestion of formulated nanofibers was performed according to the INFOGEST 2.0. protocol [51]. Samples (0.2 g) were exposed to the simulated gastric fluid (SGF) (pH = 3) and simulated intestinal fluid (SIF) (pH = 7) with dissolved gastric enzymes, prepared according to the protocol. Pepsin was weighed (New Classic ML204/01, Mettler Toledo, Greifensee, Switzerland) and dissolved into the SGF electrolyte buffer solution (enzyme activity: 2000 U mL^−1^ on the total volume of buffer used). After 120 min of the gastric phase, gastric chyme was mixed (1:1 *v*/*v*) with a previously prepared mixture of pancreatin (1 mg mL^−1^) and bile salts (2.5 mg mL^−1^) in the SIF electrolyte buffer solution. Temperature (37 °C) and homogeneous mixing (SMHS-6, Witeg Labortehnik GmbH, Wertheim, Germany) were kept constant. Sampling of the tested nanofibers and blank sample (buffer solution + enzymes) was carried out in the interval of 5–180 min, and the release of polyphenols was evaluated by TPC determination [48]. The results were expressed in mg of echinacoside equivalents per gram of nanofiber (mg eq ECH g^−1^).

#### 2.7.8. Statistical Analysis

One-way analysis of variance with Tukey’s post hoc test (significance level, α < 0.05) was employed on every set of results using the Statistica ver. 13.3 software package (TIBCO Software Inc., Palo Alto, CA, USA). ATR-FT-IR graphs and relative diameter distributions were acquired in OriginPro 2023b (ver. 10.5) (OriginLab Corporation, Northampton, MA, USA). GraphPad Prism ver. 10.1.2 (trial version; GraphPad Software, Boston, MA, USA) was employed for graphical display of rheological data. DSC thermograms were evaluated in STARe software 16.0 (Mettler Toledo, Greifensee, Switzerland).

## 3. Results and Discussion

### 3.1. Physical Properties of Polymer Solutions

A sufficient surface charge density and a relatively low force on the free liquid surface facilitate the stretching of the polymer solutions and the overall feasibility of electrospinning [52]. As can be seen for the polymer combinations that exhibited electrospinning potential (Table 3), the measured values of conductivity (1.51–2.36 mSI cm^−1^) and surface tension (30.38–42.03 mN m^−1^) correspond to the conditions for successful electrospinning performance and the production of morphologically suitable nanofibers [53,54]. Aceituno-Medina et al. (2013) [55] reported similar values of conductivity (5.4–6.7 mS cm^−1^) and surface tension (30.9–32.1 mN m^−1^) for successfully prepared electrospun nanofibers based on PUL and amaranth protein (20%) polymer blends in formic acid (95% *v*/*v*). The comparable values of conductivity and surface tension for the non-electrospinnable 15ZE (2.53 mSI cm^−1^, 27.18 mN m^−1^), 15ZE + lec (2.78 mSI cm^−1^, 39.88 mN m^−1^), and 15PUL + lec (1.79 mSI cm^−1^, 24,62 mN m^−1^) with the formulations successfully used in this study, however, indicate the great importance of the synergistic effect of the physical properties, intrinsic polymer properties, and the rheological parameters of polymer solutions, which must be taken into account.

### 3.2. Viscosity Curves and Rheological Characterization of Polymer Solutions

In addition to the physical properties, viscosity, polymer concentration, degree of molecular entanglement and elasticity of the polymer network are among the most important parameters for neutralizing Rayleigh instability and the appropriate degree of association of the polymer chains for the formation of a stable polymer jet [56].

**Figure 3:**
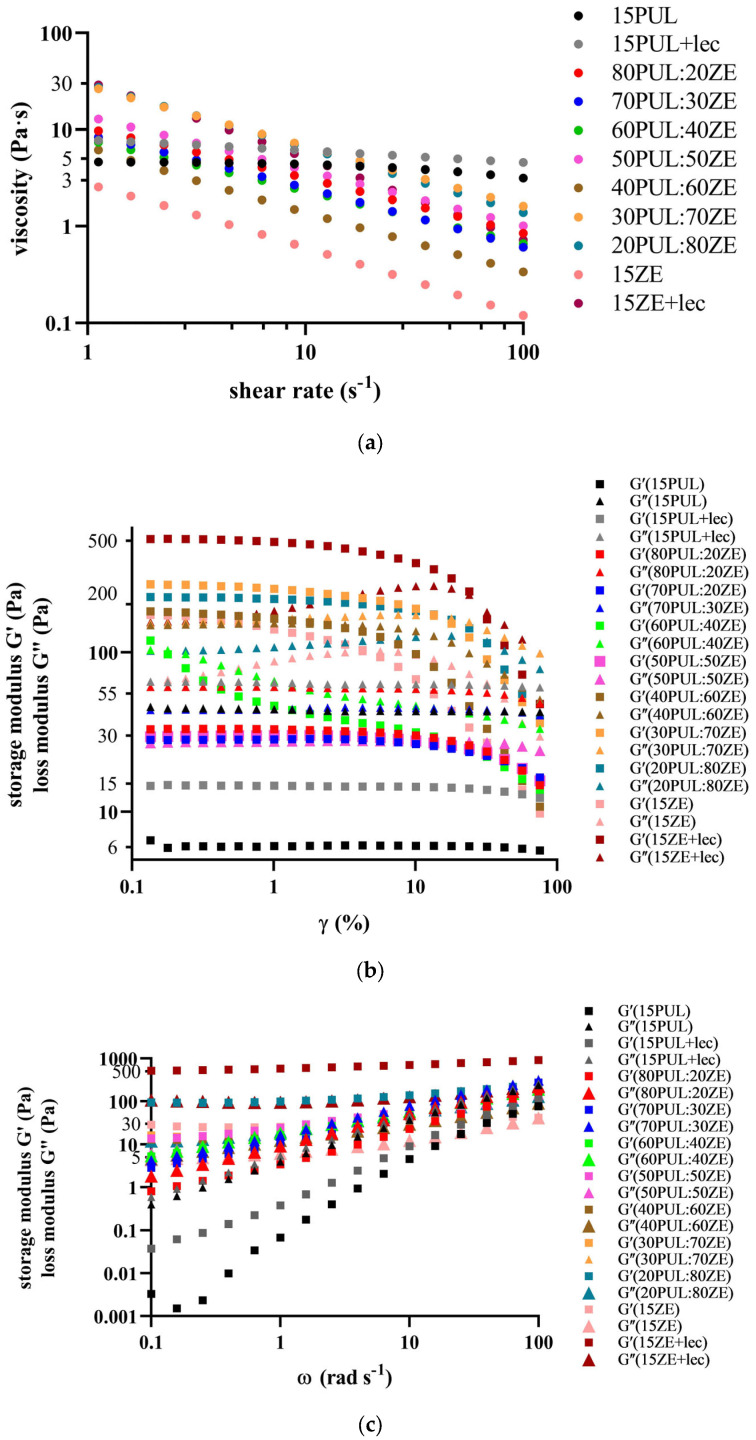
Rheological characterization of polymer solutions with determination of (**a**) absolute/apparent viscosity, (**b**) a strain amplitude sweep test, and (**c**) a frequency sweep test.

A statistically significant (*p* < 0.05) effect of increasing the amount of zein on the increase in apparent viscosity and elastic component in the binary PUL:ZE blends was observed (Table 4 and Figure 3a). Although the addition of sunflower lecithin to the zein solution significantly increased the apparent viscosity (*p* < 0.05) from 665 to 5955 mPa·s and the solution elasticity (tan δ for 15ZE: 0.37, tan δ for 15ZE + lec: 0.20) compared with pure zein by promoting the entanglement of polymer chains, the electrospinnability of the zein solution was not improved. The presence of lecithin apparently promoted the formation of hydrogen and hydrophobic intermolecular bonds between the hydrophilic polysaccharide units of pullulan and the polypeptide chains of proteins in polymer blends, thereby increasing the degree of crosslinking of the polymer chains [57,58], which was later confirmed by oscillation tests.

To further characterize the PUL:ZE pseudoplastic systems, it was necessary to determine the range of their structural stability by measuring the linear viscoelastic range (LVE) within the defined range of shear deformation (γ) (Figure 3b). Increasing the ZE content above 50% led to a significant reorganization of the structural polymer network in the form of an increase in the elastic character, i.e., the stability of the crosslinked structure over the entire frequency range (tan δ < 1) and consequently to an increase in the apparent viscosity. Luo et al. (2021) [59] also confirmed a significant increase in intermolecular interactions by introducing zein (15–30%) into a dextran-based polymer system, but also a decrease in apparent viscosity. The oscillatory measurement, which was carried out in the previously determined linear viscoelastic range, provided further insights into the crosslinking of polymer chains in the range of the applied angular frequencies (0.1–100 rad s^−1^). The introduction of lecithin into the PUL polymer solution statistically significantly (*p* < 0.05) increased the elasticity of the system, which was stable over the entire applied frequency range, compared with 15PUL, but also increased the apparent viscosity, which ultimately had a negative effect on the electrospinnability of this combination (Figure 3c). A similar trend was observed with the samples 15ZE and 15ZE + lec, which resulted in solid gel systems after extended standing time, which can be easily recognized when looking at the storage modulus curves at lower frequencies. The samples 30PUL:70ZE and 20PUL:80ZE showed an elastic character over the entire frequency range, with an additional increase in the elastic component at lower frequencies, which is a characteristic of polymer systems with a high degree of crosslinking. For 70PUL:30ZE, 60PUL:40ZE, 50PUL:50ZE, and 40PUL:60ZE, on the other hand, the “intertwining” of the curves for the systems G′ and G″ is visible, which indicates a uniform dominance of the viscous and elastic components as a function of the applied angular frequency.

### 3.3. Characterization of Electrospun Nanofibers

#### 3.3.1. The Effect of the PUL:ZE Ratio on Nanofibers’ Morphology

**Figure 4:**
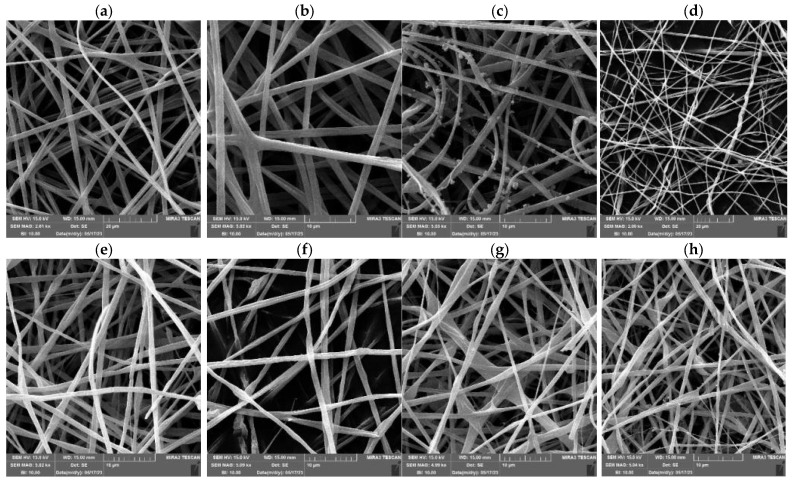
SEM micrographs of formulated nanofibers (captured at 3000–5000× magnification): (**a**) 15PUL, (**b**) 80PUL:20ZE, (**c**) 70PUL:30ZE, (**d**) 60PUL:40ZE, (**e**) 50PUL:50ZE, (**f**) 40PUL:60ZE, (**g**) 30PUL:70ZE, and (**h**) 20PUL:80ZE.

SEM micrographs showed that all PUL:ZE formulations led to successfully produced nanofibers with a ribbon-shaped structure and a uniaxial orientation (Figure 4). In addition, a less uniform structure can be observed in the 30PUL:70ZE and 20PUL:80ZE samples, with occasional thinned or thickened areas in the structure itself. The presence of nanoparticles on the nanofibrous structures of 70PUL:30ZE can be explained by the short-term destabilization of the polymer jet during the electrospinning process using the needle system, which resulted in insufficiently rapid evaporation of the solvent, forming globules/particles of incompletely electrospun solution. In the study of Deng et al. (2018) [60], nanofibers based on binary collagen and zein systems resulted in crosslinked and alternately present ribbon-shaped nanofibers with a flat surface. Such a morphology is a consequence of the collapse due to the influence of atmospheric pressure during the instantaneous evaporation of the solvent, causing ellipticity and a flat surface, and the bending of the nanofibers into a ribbon-shaped shape due to the influence of electrostatic instability [61].

**Figure 5:**
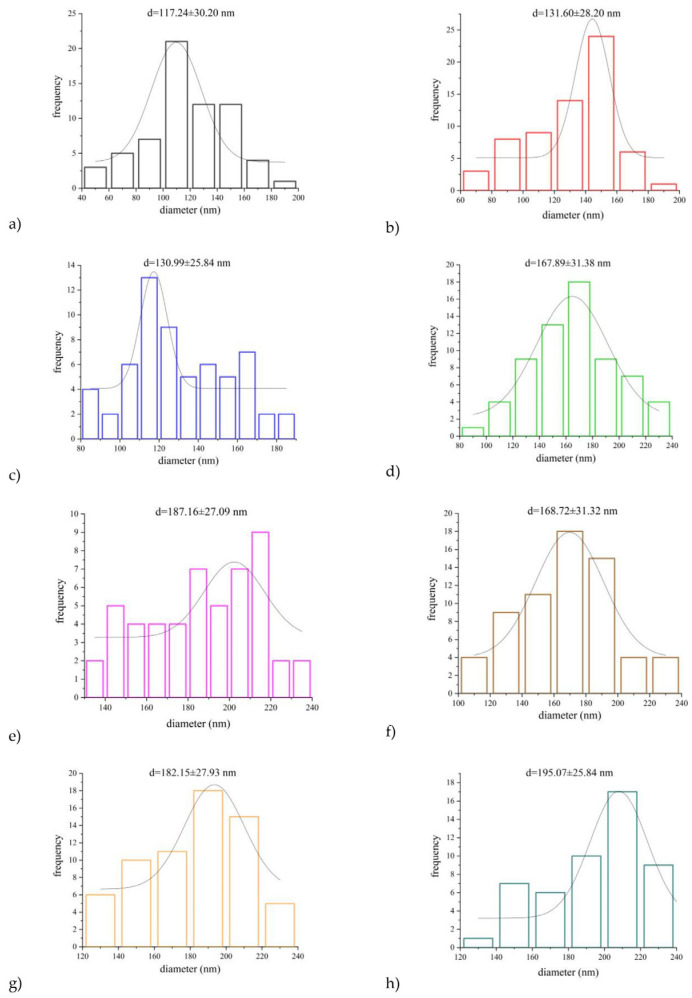
Relative diameter distribution with average diameter size in formulated nanofibers: (**a**) 15PUL, (**b**) 80PUL:20ZE, (**c**) 70PUL:30ZE, (**d**) 60PUL:40ZE, (**e**) 50PUL:50ZE, (**f**) 40PUL:60ZE, (**g**) 30PUL:70ZE, and (**h**) 20PUL:80ZE.

The *average diameter* for the 15PUL and electrospinnable PUL:ZE solutions ranged between 117,24 and 195,07 nm, whereby increased diameter positively correlated with the higher zein proportion (Figure 5). It is known that an increase in the proportion of polymers with a higher molecular weight and the degree of crosslinking of the polymer chains leads to an increased average diameter of the nanofibers [54,62]. For instance, Deng et al. (2018) [60] also confirmed a positive correlation between apparent viscosity and average nanofiber diameter during electrospinning of binary systems of collagen and zein. A similar morphology was also observed in the work of Wang et al. (2019) [63], and a significant influence of the type of solvent on the diameter distribution, viscosity increase, and zein concentration on the improvement of the morphology of the nanofibers, without the presence of particles, was confirmed. However, the only moderately positive correlation (r = 0.51) between the average diameter and apparent viscosity for PUL:ZE nanofibers indicates the importance of some other factors influencing the diameter of the nanofibers.

**Figure 6:**
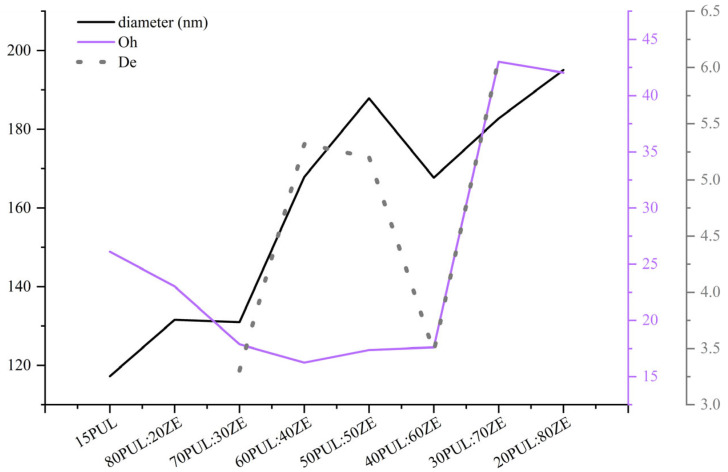
The correlations among the average diameter size, Ohnesorge number, and Deborah number in nanofiber samples.

The dependence of apparent viscosity, surface tension, the density of polymer solutions, and droplet diameter on morphological properties can be defined by the dimensionless *Oh* number—a quantitative measure of the viscous component in a solution that represents the ratio of the dissipation of intrinsic viscosity and surface tension forces. It can be used to characterize the break-up of the polymer. No positive correlation between the average diameter and the *Oh* number (r = 0.35) was observed for the PUL:ZE-based nanofibers produced, following the trend of a weak correlation with the measured apparent viscosity values for the same polymer solutions (Figure 6). However, a strong positive correlation (r = 0.75) was observed between the diameter and the elastic component of the polymer solutions, quantitatively integrated into the *De* number. Gupta et al. (2015) and Flores-Hernandez et al. (2020) [64,65] also reported a positive correlation between *De* and fiber diameter, highlighting the importance of considering not only viscosity and polymer concentration optimization, but also the elastic properties that govern nanofiber formation.

#### 3.3.2. Characterization of Functional Groups and Their Interactions

**Figure 7:**
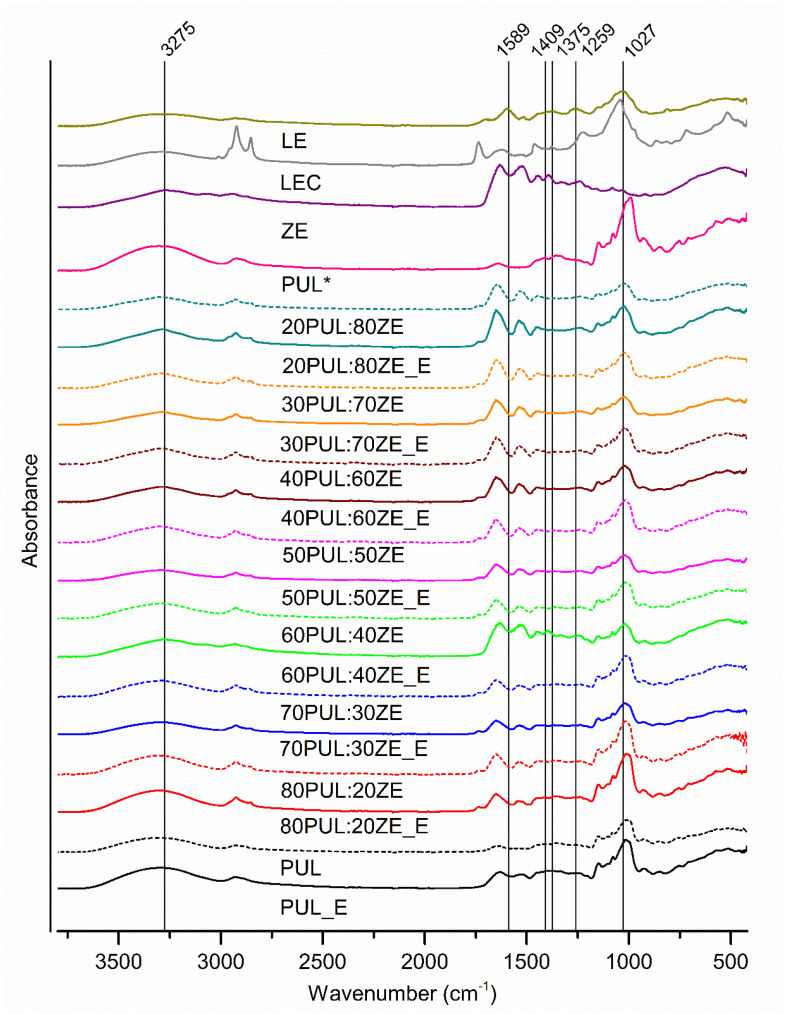
ATR-FT-IR spectra of the formulated nanofibers. LEC, sunflower lecithin; LE, lyophilized, non-encapsulated extract; PUL*, pullulan; ZE, zein; samples without „_E”, nanofibers without extract; samples marked with „_E”, nanofibers with extract.

The analysis of the ATR-FT-IR spectra confirmed the presence of physicochemical interactions between the polymer carriers and the polyphenolic compounds of the MG extract (Figure 7).

The FT-IR spectrum for the MG extract revealed a larger number of characteristic absorption bands for polyphenolic compounds in the wavenumber range of 900–1800 cm^−1^. Several overlapping bands with an absorption maximum at 1027 cm^−1^ are probably due to the stretching of symmetric C-O-C and C-O bonds, confirming the presence of glycosidic bonds between hydroxytyrosol and glycones in phenylethanoid glycosides. A broad band with pronounced intensity at ~3500 cm^−1^ confirms the presence of hydroxyl groups involved in intra- and intermolecular bonding. The detected bands at 1259 cm^−1^ (C-O), 1375 cm^−1^ (C-H; O-H), 1589 cm^−1^ (C-C; C=C), 2930 cm^−1^ (C-H), and 3275 cm^−1^ (O-H) can be attributed to the aromatic structures of the polyphenolic compounds but also to other plant metabolites with the same profile of functional bonds. Pullulan (PUL*) showed characteristic absorption bands at 753 cm^−1^, 846 cm^−1^, and 929 cm^−1^, indicating the presence of α-(1-4)-glycosidic bonds between α-D-glucopyranose units within the repeating maltotriose unit, i.e., α-(1-6)-D-glycosidic bonds between maltotriose units [66]. Also, the absorption maxima at 1077 cm^−1^, 1147 cm^−1^, and 1206 cm^−1^ are attributed to C-O-C stretching, while the maxima at 1413 cm^−1^, 1354 cm^−1^, and 2925 cm^−1^ signify C-H bending and C-H stretching, respectively [66,67]. The presence of OH functional groups originating from theformed hydrogen bonds was also observed at 3300 cm^−1^ [68]. Zein (ZE) also led to characteristic absorption bands for proteins in the absorption ranges of 1644–1651 cm^−1^, 1515–1530 cm^−1^, and 1238–1447 cm^−1^, corresponding to the functional groups of Amide I, Amide II, and Amide III, respectively. Consecutive bands of lower intensity between 2872 and 2957 cm^−1^ correspond to C-H stretching, while a broad band with a maximum at 3292 cm^−1^ is due to bonds within the Amide A group (N-H stretching) [60]. Sunflower lecithin (LEC) showed maximum peaks corresponding to the ester bond between fatty acid and glycerol (stretching of the carbonyl group at 1735 cm^−1^), phosphate groups (1224 cm^−1^), and C=C vibrations originating from the unsaturated fatty acid (smaller and partially merged maxima at 1654 cm^−1^ and 1618 cm^−1^) [69]. In addition, dominant C-H bonds were detected in phospholipid structures (1459 cm^−1^, 2922 cm^−1^, and 2853 cm^−1^). The maximum at 1042 cm^−1^ can be associated with the presence of C-O stretching and C-O-H bending [70]. The intense absorption at 3293 cm^−1^ indicates the presence of intermolecularly bound O-H groups, probably within the structure of phosphatidylinositol, the dominant phospholipid (PL) in the overall composition of lecithin.

Comparing the formulated nanofibers with and without incorporated polyphenolic extract, characteristic absorption bands for the extract and the polymers as well as an increase in the intensity of the Amide I functional group (1750–1600 cm^−1^) at a higher ZE content can be observed in all nanofiber samples. A decrease in the intensity of the bands was also observed in the investigated nanofibers with extract compared with nanofibers without extract, which is an indicator that electrostatic, steric, and hydrophobic interactions formed [71,72]. Electrostatic interactions, such as hydrogen interactions, play a key role in the formation of polysaccharide and protein complexes due to the large number of differently charged functional groups of proteins (amino acid side branches, amino and carboxyl groups) as a function of pH, with the proportion of biopolymers and the amount of charged groups present determining their strength [73]. The shift of the detected maximum peak in all nanofibers without MG extract from 2931 cm^−1^ to 2855 cm^−1^ and between 1238 and 1243 cm^−1^ with respect to LEC is probably the result of interactions between the polymer and LEC, confirming the role of sunflower lecithin as a stabilizer between the hydrophilic pullulan and the relatively hydrophobic structure of zein. Accordingly, the interactions between the functional groups LE, PUL, ZE, and LEC in the nanofibers were investigated in the context of the FT-IR spectrum of LEC. It is known that polar groups of surfactants interact with charged amino acid groups of globular proteins, while they can also form hydrophobic interactions via the alkyl chain [74]. In the case of the PUL:ZE blends enriched with MG extract, shifts in the maximum of the absorption bands from 1027 cm^−1^ to 1012–1022 cm^−1^ and shifts to a lower wavenumber in the range of 1735 to 1535 cm^−1^ indicate that electrostatic interactions formed between the functional groups of the polyphenolic compounds of the extracts, the polymers, and the LEC. Zein primarily creates hydrogen interactions with the hydroxyl groups of the polysaccharides via glutamine [75], while it also forms hydrophobic interactions with the polyphenolic compounds, depending on their polarity.

#### 3.3.3. The Effect of Polyphenolic Extract on Secondary Conformation Change

**Figure 8:**
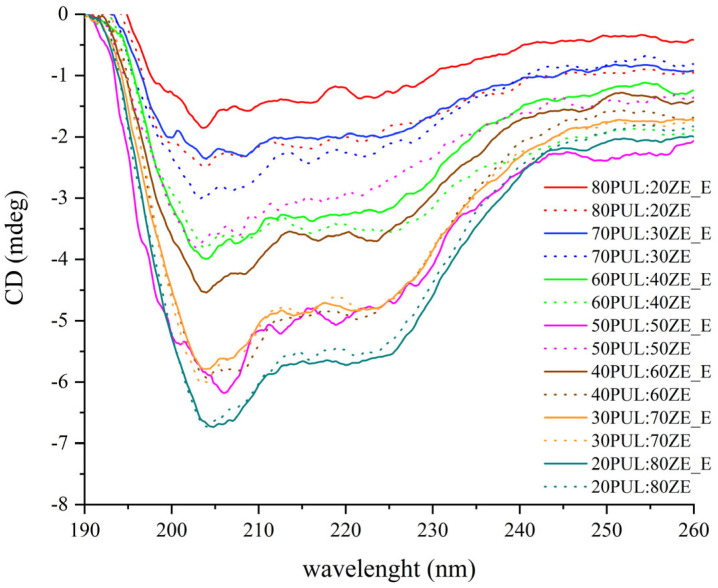
CD spectra of formulated PUL:ZE nanofibers. Samples without „_E”, nanofibers without extract; samples marked with „_E, nanofibers with extract.

Circular dichroism (CD) analysis of the nanofibers enriched with MG polyphenolic extract confirmed the change in ellipse intensity and the shift of the detected peak maximum compared with the empty nanofibers (Figure 8). For all PUL:ZE samples, two negative maxima are observed in the spectra: a more intense one between 204–206 nm, indicating a random coil, and a broader and less pronounced one between 218 and 224 nm. In particular, the absorption maximum at 222 nm of the 70PUL:30ZE, 40PUL:60ZE, and 30PUL:70ZE samples indicates the presence of an α-helical conformation [76], while the CD spectra for 50PUL:50ZE and 20PUL:80ZE indicate the presence of a ß-sheet within the structure (218–220 nm) [77].

These spectral changes indicate a decrease or increase in the proportion of random turns and thus a more open, interactive protein structure, or α-helices and ß-sheets for a more stable and closed protein conformation. Only for the samples 30PUL:70ZE_E and 20PUL:80ZE_E was an overlap of the spectra with the corresponding samples of nanofibers without extract observed, indicating an unchanged secondary conformation of the protein structure.

In the previous part of the discussion on the characterization of encapsulations by ATR-FT-IR analysis, the nature of the interactions between proteins and polyphenols was mentioned. This is a predominantly non-covalent type of interaction that includes hydrophobic interactions and electrostatic interactions such as hydrogen bonding. They largely depend on the properties of the proteins (conformation, stereospecificity of binding sites, functional groups of neighboring amino acids) and the polyphenolic compounds (molecular mass, degree of polymerization, conformational flexibility, etc.) [78,79,80]. MG extract is rich in phenylethanoid glycosides consisting of aromatic structures (caffeic acid and hydroxytyrosol) and polar sugar units, which enable the formation of hydrophobic interactions and hydrogen bonds within the PUL–LEC–ZE system. In the presence of lecithin, PUL with its hydroxyl and ionizing groups can additionally stabilize hydrophobic proteins and interact with the sugar units of phenylethanoid glycosides, while proteins can simultaneously interact with alkyl chains and the phosphate group of phospholipids as well as via more polar amino acid side branches and with polyphenols. The change in secondary conformation due to the interaction of polyphenolic compounds and proteins, i.e., the increase in the proportion of the α-helix compared with the random coil in the interaction of soy proteins and tea polyphenols, was also found in the work of Ge et al. (2021) [81]. An ordered and stable secondary conformation was also maintained when curcumin was encapsulated in zein nanoparticles [77]. However, Liu et al. (2017) observed an increased proportion of the more open, less stable structure of zein (an increase in the proportion of random coil) when the conjugate of quercetagetin and zein was formulated [76]. Similar results were obtained by Zhao et al. (2020) when investigating the interactions between phenolics (gallic acid and tannic acid) and proteins (casein and collagen), finding that the ordered secondary conformation was disrupted [82].

#### 3.3.4. Thermal Properties of Electrospun Nanofibers

**Figure 9:**
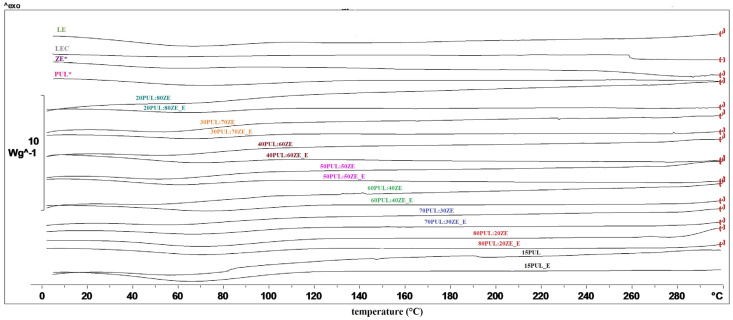
DSC thermograms of formulated PUL:ZE nanofibers. LEC, sunflower lecithin; LE, lyophilized, non-encapsulated extract; PUL*, pullulan; ZE*, zein; samples without „_E”, nanofibers without extract; samples marked with „_E”, nanofibers with extract.

Thermal analyses of polymer carriers for encapsulation are necessary to determine possible endothermic or exothermic changes in a given temperature range, which is crucial for evaluating the possibility of their use in matrices subjected to certain heat treatments. In order to determine the influence of the MG extract on the thermal properties of the polymers used, the DSC technique was used. According to the obtained thermograms, no phase transitions occurred in the analyzed polymers (PUL and ZE), and the glass transition temperature was not recorded (Figure 9). The same observation can be made for all nanofiber formulations with and without extract, indicating consistent stability within the applied temperature range.

As can be seen in Table 5, the endothermic peaks at 56–75 °C for all nanofibers without extract (26.83–123.52 J g^−1^) and the nanofibers enriched with extract (5.95–99.66 J g^−1^), with the exception of LEC, are related to the evaporation of water from the samples [16,83]. Shao et al. (2018) also observed no significant changes in the thermal properties of pullulan in the presence of a polyphenolic extract of *C. sinensis* [84]. On the other hand, Ren et al. (2022) successfully demonstrated the improved thermal stability of covalent conjugates of zein and resveratrol as innovative encapsulated systems with improved technological properties [85].

#### 3.3.5. Encapsulation Efficiency of Electrospun Nanofibers

**Figure 10:**
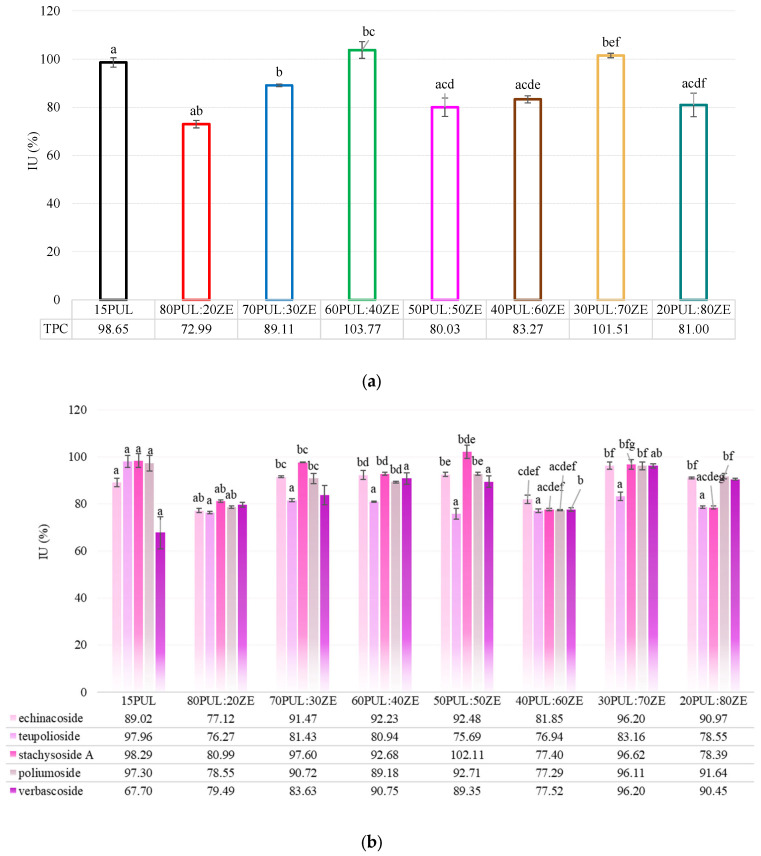
(**a**,**b**) Encapsulation efficiency (%) of total phenolic content and phenylethanoid glycosides (echinacoside, teupolioside, stachysoside A, poliumoside and verbascoside) in nanofiber formulations. Values marked with the same letter are statistically significant (*p* < 0.05).

The PUL:ZE delivery matrix generally proved to be suitable for the retention of studied polyphenols from the MG extract (EE for TPC: 72.99–103.77%) (Figure 10a). However, depending on the PUL and ZE ratio, some formulations proved to be less efficient for the entrapment of individual PGs, i.e., echinacoside (EE for 80PUL:20ZE: 77.12%), teupolioside (EE for all PUL:ZE samples: 76.27–80.94%), Stachysoside A (EE for 80PUL:20ZE: 80.99%; EE for 40PUL:60ZE: 77.40%; 20PUL:80ZE: 78.39%) and poliumoside (EE for 80PUL:20ZE: 78.55%; EE for 40PUL:60ZE: 77.29%), compared with the PUL carrier (Figure 10b). However, 15PUL yielded the lowest EE for verbascoside (67.70%). A medium positive Pearson correlation between the EE of individual PGs and the TPC (r = 0.51–0.65, depending on the compound analyzed) was found for all tested formulations, except for verbascoside (r = 0.14). These results could indicate the presence of a strong affinity of other polyphenols in the MG extract that are not the focus of this study, e.g., flavonoid glycosides, to bind the tested polysaccharide–protein matrix. Although there is a great lack of studies on the application of electrospinning of phenylethanoid glycosides, recent studies have mainly used other nanosystems for their encapsulation. For example, Li et al. (2016) encapsulated a concentrate of phenylethanoid glycosides from *Cistanche* spp. in the form of liposomes using soya phospholipids, poloxamer 188, and sodium deoxycholate, resulting in a low encapsulation efficiency (26.58–53.90%) [86]. On the other hand, Wu et al. (2023) formulated highly effective verbascoside lipid nanocapsules (EE: 85%) using the reverse micelle technique and achieved prolonged storage stability [87]. The high EE can be attributed to the realized electrostatic and hydrophobic interactions between the polymer carriers and the polyphenolic compounds of the extract, which have already been explained in Section 3.3.2.

#### 3.3.6. Zeta Potential of Electrospun Nanofibers

**Figure 11:**
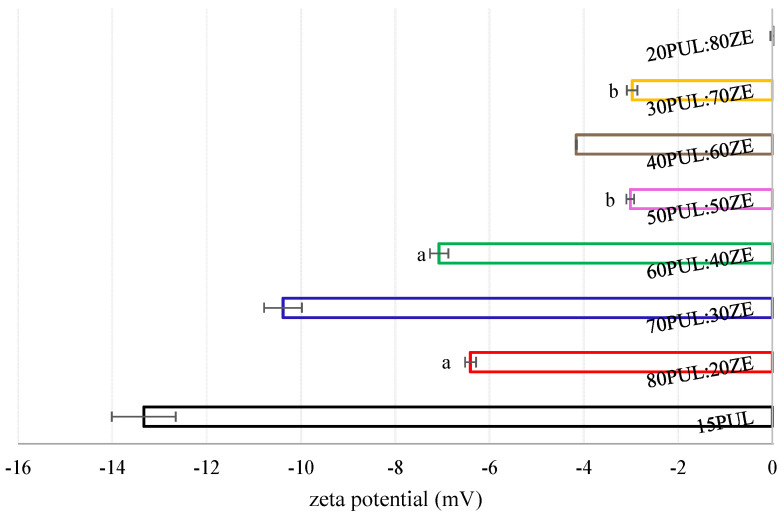
Zeta potential of formulated PUL:ZE nanofibers. Values marked with the same letter are statistically not significant (*p* > 0.05).

The zeta potential is a physical property of colloidal systems and refers to the presence of electrostatic charges on the surface of particles dispersed in the liquid phase. It is an indicator of the stability of the dispersion [88]. In this study, an increased amount of zein in binary polymer systems with pullulan led to a statistically significant decrease in electrostatic repulsion (*p* < 0.05) (Figure 11). The results revealed the tendency of the investigated PUL:ZE formulations to agglomerate in aqueous acidic environments, leading to the formation of unstable suspensions. The opposite results were obtained in other studies, in which nanofibers based on zein-encapsulated carotenoid microemulsions [89] and geraniol-loaded liposomes [90] in the presence of lecithin led to stable dispersions, i.e., zeta potentials of −29.73 mV and −38.30 mV, which was explained by the presence of free sulphate groups of phospholipids on the surface of nanoliposomes. In addition, Sun et al. (2018) also confirmed the positive influence of the addition of lecithin on increasing the zeta potential and hydrophobicity of the particles’ surface within the whey protein system, which is explained by the binding ability of surfactants to proteins, which results in unfolding of secondary protein conformation [91]. The redistribution and re-formation of intra- and intermolecular electrostatic and hydrophobic interactions allow a greater availability of negatively charged amino acids on the surface, increasing the electrostatic repulsions along with the negatively charged sulphate groups of the lecithin. In this case, it is possible that in the tested PUL:ZE systems, positively charged amino acids interacted with the free sulphate groups of the lecithin due to a partial disruption of the secondary structure of the protein, thus influencing the “neutralization” of the surface charge of the nanofibers, i.e., a reduction in the zeta potential.

#### 3.3.7. In Vitro Kinetic Release of Polyphenols

**Figure 12:**
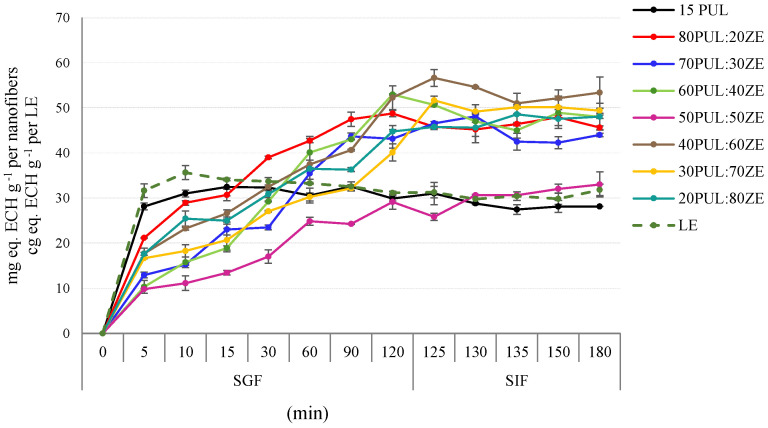
Kinetic release of total phenolic content in simulated in vitro gastrointestinal conditions from nanofiber delivery systems. LE, lyophilized extract.

The results of the kinetics of simulated in vitro digestion generally indicate the good potential of PUL:ZE polymer blends to bind the polyphenolic compounds and allow their prolonged release in the simulated gastrointestinal fluid (Figure 12). With the exception of the sample 15PUL, which followed accelerated and complete TPC release during the first 10 min of SGF phase, the remaining tested samples resulted in a relatively sustained release of polyphenols during the 120 min of the SGF phase. However, the formulations 70PUL:30ZE, 60PUL:40ZE, and 40PUL:60ZE showed a slight decrease in TPC in SIF after 125 min. The 50PUL:50ZE binary mixture showed the best release profile in the SGF phase, with polyphenols also being retained in the SIF phase over 60 min. This effect is likely due to structural changes in the protein induced by the polyphenolic extract, particularly the increased random coil and β-sheet content in 50PUL:50ZE_E compared with empty nanofibers. Non-covalent polyphenol binding partially unfolded the zein’s secondary structure, exposing hydrophobic residues and concurrently prolonging polyphenol release in the simulated gastrointestinal environment.

Some previous research also indicates a number of benefits when proteins with a hydrophobic profile are combined with hydrophilic polysaccharides such as xanthan gum, gum arabic, and pullulan to improve the physicochemical properties and prolonged release of curcumin, a polyphenolic compound from the extract of *Ruta chalepensis*, i.e., silybin as an active component of silymarin from nanosystems [92,93,94]. By introducing polysaccharides with ionizable groups into the zein solution, desirable electrostatic interactions are formed, which stabilize the complexes and reduce the aggregation and precipitation of zein in a polar environment due to its distinct hydrophobic nature, thereby stabilizing the nanosystems in a wider pH range, while improving the encapsulation efficiency and modifying the release kinetics of the active components [95].

## 4. Conclusions

The aim of this study was to formulate nanodelivery systems for mountain germander extract (MG) by a sustainable electrospinning process of pullulan/zein suspensions stabilized with sunflower lecithin. The effect of physical and viscoelastic properties of polymer solutions on the morphology of the nanofibers was analyzed, as well as the influence of the pullulan/zein ratio on the functional properties of the developed nanofibers. The rheological characterization of PUL:ZE suspensions indicate the importance of optimizing the viscoelastic properties of the polymer blend in order to achieve an appropriate morphology and diameter when the other requirements for electrospinning are met. The addition of zein increased the elastic component, as reflected by a higher Deborah number, and led to larger nanofiber diameters. Regardless of the PUL:ZE ratio, all formulations showed promising potential for the encapsulation of phenylethanoid glycosides from the MG extract (EE: >73%). FT-IR analysis and CD spectra showed the high affinity of the polysaccharide–protein–lecithin carrier matrix for intermolecular binding of polyphenols, which was later confirmed by the sustained release of polyphenols during simulated in vitro digestion. The significant reduction (*p* < 0.05) in electrostatic surface charge with increasing ZE content suggests reduced solubility of the carrier systems in aqueous media, highlighting the suitability of the formulated PUL:ZE nanofibers for incorporation into semi-solid or solid food matrices such as gummies, powdered nutraceuticals, or nutritional bars. Possible approaches for further investigations and improvements could be to develop a predictive ANN model that takes into account the polymer properties and process parameters as key variables for successful electrospinning, focusing on the viscoelastic properties of the polymer blend.

## Figures and Tables

**Table 1:** Polyphenolic profile of mountain germander extract.

Echinacoside (g L^−1^)	Teupolioside (g L^−1^) *	Stachysoside A (g L^−1^) *	Poliumoside (g L^−1^) *	Verbascoside (g L^−1^)	TPC * (g L^−1^)
2.38 ± 0.08	0.69 ± 0.02	1.30 ± 0.64	0.92 ± 0.45	0.60 ± 0.29	8.92 ± 0.03

* Results were expressed in grams of echinacoside equivalents per liter of extract.

**Table 2:** Pullulan/zein formulations for the production of electrospun nanofibers.

Pullulan (PUL) (%)	Zein (ZE) (%)	Lecithin (lec) (%)	Sample Abbreviation	Electrospinnability Potential	Morphology
100	0	-	15PUL	-	nanofibers
100	0	3	15PUL + lec	+	/
80	20	3	80PUL:20ZE	+	nanofibers
70	30	3	70PUL:30ZE	+	nanofibers
60	40	3	60PUL:40ZE	+	nanofibers
50	50	3	50PUL:50ZE	+	nanofibers
40	60	3	40PUL:60ZE	+	nanofibers
30	70	3	30PUL:70ZE	+	nanofibers
20	80	3	20PUL:80ZE	+	nanofibers
0	100	-	15ZE	-	/
0	100	3	15ZE + lec	-	/

**Table 3:** Conductivity and surface tension of the polymer solutions.

Sample	σ (mSI cm^−1^)	γ (mN m^−1^)
extract	2.02 ± 0.01	36.19 ± 0.28
acidified extract ^1^	1.33 ± 0.01	37.79 ± 0.06
15PUL	1.51 ± 0.01 ^a^	35.11 ± 0.84 ^a^
15PUL + lec	1.79 ± 0.00 ^b^	24.62 ± 0.23 ^b^
80PUL:20ZE	1.67 ± 0.03 ^c^	30.38 ± 0.40 ^c^
70PUL:30ZE	1.65 ± 0.01 ^cd^	31.39 ± 0.95 ^cd^
60PUL:40ZE	1.57 ± 0.01 ^acd^	31.49 ± 1.08 ^cde^
50PUL:50ZE	1.91 ± 0.01 ^be^	31.23 ± 0.40 ^cde^
40PUL:60ZE	1.94 ± 0.00 ^e^	34.72 ± 1.20 ^a^
30PUL:70ZE	1.82 ± 0.00 ^be^	42.03 ± 1.45 ^f^
20PUL:80ZE	2.36 ± 0.06	40.84 ± 2.82 ^g^
15ZE	2.53 ± 0.10	27.18 ± 0.48 ^b^
15ZE + lec	2.78 ± 0.03	39.88 ± 0.24 ^fg^

^1^ acidified = extract concentrated 20-fold, further diluted in glacial acetic acid in a 1:1 ratio (*v*/*v*). Values marked with the same letter within a column are not statistically significant (*p* > 0.05).

**Table 4:** Storage modulus (G′), loss modulus (G″), and loss factor (tan δ) for the results of apparent viscosity.

Sample	G′ _LVE_ (Pa)	G″ _LVE_ (Pa)	tan δ _LVE_	Viscosity at 25 s^−1^ (mPa s)
15PUL	4.63 ± 0.10 ^a^	36.90 ± 0.10 ^a^	7.97 ± 0.16	4129.55 ± 12.85
15PUL + lec	9.70 ± 0.68 ^ab^	47.77 ± 1.65 ^ab^	4.94 ± 0.18	6169.65 ± 18.75 ^a^
80PUL:20ZE	18.73 ± 4.02 ^ac^	44.00 ± 8.15 ^abc^	2.37 ± 0.07	3367.55 ± 8.05 ^b^
70PUL:30ZE	47.03 ± 1.81 ^d^	81.68 ± 2.05 ^d^	1.74 ± 0.02	2645.9 ± 8.6 ^c^
60PUL:40ZE	54.22 ± 0.35 ^de^	57.82 ± 0.78 ^abcde^	1.07 ± 0.01 ^a^	2405.15 ± 61.15 ^cd^
50PUL:50ZE	52.29 ± 8.46 ^def^	41.12 ± 6.92 ^abef^	0.79 ± 0.01 ^ab^	2525.95 ± 49.55 ^cde^
40PUL:60ZE	43.57 ± 1.62 ^defg^	30.68 ± 1.35 ^abcfg^	0.70 ± 0.01 ^abc^	2749.4 ± 39.7 ^bcde^
30PUL:70ZE	43.57 ± 4.62 ^defgh^	30.68 ± 1.35 ^abcfgh^	0.70 ± 0.01 ^abcd^	7362.25 ± 10.05 ^f^
20PUL:80ZE	138.43 ± 0.16	52.91 ± 0.44 ^abcefgh^	0.38 ± 0.00 ^bcdef^	7075.3 ± 71.2 ^f^
15ZE	32.60 ± 1.66 ^bcdefgh^	12.15 ± 0.17 ^gh^	0.37 ± 0.01 ^bcdef^	665 ± 18.8
15ZE + lec	696.91 ± 10.09	137.60 ± 9.19	0.20 ± 0.02 ^f^	5955.5 ± 31.25 ^a^

G′, G″, and tan δ were determined by a frequency sweep test at 10 rad s^−1^. Values marked with the same letter within a column are not statistically significant (*p* > 0.05).

**Table 5:** DSC results of evaporating temperature and enthalpy changes for the tested polymers, lyophilized extract, and nanofibers.

Sample	T_evp_ (°C)	∆H (J g^−1^)
15PUL	68.77	123.52
15PUL_E	65.90	99.66
80PUL:20ZE	71.22	74.28
80PUL:20ZE_E	72.37	62.94
70PUL:30ZE	66.42	32.68
70PUL:30ZE_E	75.22	44.91
60PUL:40ZE	62.73	73.85
60PUL:40ZE_E	65.44	56.46
50PUL:50ZE	72.10	31.94
50PUL:50ZE_E	72.28	27.99
40PUL:60ZE	59.94	26.83
40PUL:60ZE_E	72.91	19.24
30PUL:70ZE	56.50	34.32
30PUL:70ZE_E	68.18	5.95
20PUL:80ZE	63.19	27.23
20PUL:80ZE_E	66.24	12.56
PUL*	68.93	46.75
ZE*	63.08	25.30
LEC	-	-
LE	64.91	123.69

LEC, sunflower lecithin; LE, lyophilized, non-encapsulated extract; PUL*, pullulan; ZE*, zein; samples without „_E”, nanofibers without extract; samples marked with „_E”, nanofibers with extract.

## Data Availability

The original contributions presented in this study are included in the article. Further inquiries can be directed to the corresponding author.
